# Metabolomic Profiling in Cattle Experimentally Infected with *Mycobacterium avium* subsp. *paratuberculosis*

**DOI:** 10.1371/journal.pone.0111872

**Published:** 2014-11-05

**Authors:** Jeroen De Buck, Rustem Shaykhutdinov, Herman W. Barkema, Hans J. Vogel

**Affiliations:** 1 Department of Production Animal Health, Faculty of Veterinary Medicine, University of Calgary, Calgary, Alberta, Canada; 2 Biochemistry Research Group, Department of Biological Sciences, Faculty of Sciences, University of Calgary, Calgary, Alberta, Canada; Universita di Sassari, Italy

## Abstract

The sensitivity of current diagnostics for Johne's disease, a slow, progressing enteritis in ruminants caused by *Mycobacterium avium* subsp. *paratuberculosis (*MAP*)*, is too low to reliably detect all infected animals in the subclinical stage. The objective was to identify individual metabolites or metabolite profiles that could be used as biomarkers of early MAP infection in ruminants. In a monthly follow-up for 17 months, calves infected at 2 weeks of age were compared with aged-matched controls. Sera from all animals were analyzed by ^1^H nuclear magnetic resonance spectrometry. Spectra were acquired, processed, and quantified for analysis. The concentration of many metabolites changed over time in all calves, but some metabolites only changed over time in either infected or non-infected groups and the change in others was impacted by the infection. Hierarchical multivariate statistical analysis achieved best separation between groups between 300 and 400 days after infection. Therefore, a cross-sectional comparison between 1-year-old calves experimentally infected at various ages with either a high- or a low-dose and age-matched non-infected controls was performed. Orthogonal Projection to Latent Structures Discriminant Analysis (OPLS DA) yielded distinct separation of non-infected from infected cattle, regardless of dose and time (3, 6, 9 or 12 months) after infection. Receiver Operating Curves demonstrated that constructed models were high quality. Increased isobutyrate in the infected cattle was the most important agreement between the longitudinal and cross-sectional analysis. In general, high- and low-dose cattle responded similarly to infection. Differences in acetone, citrate, glycerol and iso-butyrate concentrations indicated energy shortages and increased fat metabolism in infected cattle, whereas changes in urea and several amino acids (AA), including the branched chain AA, indicated increased protein turnover. In conclusion, metabolomics was a sensitive method for detecting MAP infection much sooner than with current diagnostic methods, with individual metabolites significantly distinguishing infected from non-infected cattle.

## Introduction

*Mycobacterium avium* subsp. *paratuberculosis* (MAP) is the etiological agent of Johne's disease (JD), a debilitating chronic enteritis in ruminants. It is well known that JD causes great economic losses on dairy farms, due to decreased milk production, premature culling, and reduced carcass value. Infected dairy cows slowly evolve from stage 1, during which no effects of infection can be observed or detected diagnostically by routine diagnostic tests, through stage 2, when a portion start to shed the bacteria and develop a detectable immune response, to stages 3 and 4, which are characterized by increasingly severe clinical symptoms.

To control the spread of MAP, test-based culling is typically recommended. However, a major challenge with controlling JD is the inability to detect MAP-infected cattle prior to them becoming infectious (fecal shedding), which can take several years to occur. Current diagnostic tests, such as fecal culture, fecal PCR and ELISA, have high test sensitivities for detecting cattle in later stages of infection, but very low test sensitivities for detection in the early subclinical stages, when shedding levels and antibody titers are low or non-existent [1]–[3]. Consequently, these tests produce many false-negative results in subclinical cattle, making interpretation and utilization challenging in the majority of cases. As a result, test and cull strategies are largely ineffective; they fail to control spread of the disease within infected herds, with and increased risk to introduce the disease in uninfected herds. Therefore, diagnostics that can detect and early infection are urgently needed.

In recent years, effects of bacterial infections on the host have been studied with increasing depth and scope, using advanced techniques, comprehensive platforms and analytical methods. In that regard, transcriptomics and proteomics have been used to study expression of genes and proteins in order to identify biomarkers and to place them in the context of specific pathways which fit the etiology and progression of a disease. From these studies, using proteomic and transcriptomic analyses, several putative biomarkers for early infection with MAP have been proposed [4]–[8]. Unfortunately, apparently none of these biomarkers have been validated on cattle of varying ages or in various stages of diseases to determine their true sensitivity and specificity.

The emerging field of metabolic profiling (i.e. metabolomics) involves identification and quantification of numerous low molecular weight compounds in biological fluid samples. Metabolomics provides a functional alternative or complement to the above-mentioned techniques, as it measures chemical phenotypes that are the net result of all activity on the transcriptome and proteome levels; therefore, it provides an integrated and reductive profile of the status of the test subject. Recent reports have demonstrated the potential of metabolomics to improve current clinical microbiology diagnostic methods [9], [10].

Clinical JD is very likely associated with a complicated array of chemical reactions and metabolites that stem from a diverse set of metabolic pathways associated with inflammation [11] and infection. However, we hypothesized that due to changes in gut function and metabolism caused by subclinical MAP infection, a characteristic pattern of metabolites would also be detectable. Therefore, the aim of this study was to determine whether metabolic profiling could be a reliable tool to detect early MAP infection in dairy calves. For this purpose, dairy calves were experimentally infected with MAP at various ages and with two doses of the pathogen, and a ^1^H NMR spectroscopy-based metabolomics approach was used to identify a characteristic metabolic ‘biomarker pattern’ in subclinically infected calves. The ability to discover early MAP infection in calves by metabolic profiling of sera has apparently been reported. First, a longitudinal follow-up of infected and non-infected cattle was done to identify how early after infection these calves could be discriminated. Next, non-infected cattle were compared with infected cattle at a fixed age (1 year old) in a cross-sectional analysis.

## Methods

### Experimental infection

Experimental details were identical to the infection study described in Mortier *et al*. 2013 [12]. Briefly, male Holstein-Friesian calves (n = 35) were obtained from MAP-free or low prevalence dairy herds on the day they were born. To minimize the risk of including calves that had acquired intra-uterine MAP infection, calves were collected only from the 16 herds that yielded negative pooled (*n* = 5) fecal samples (decontaminated and prepared for culture according to manufacturer's instructions; para-JEM, TREK Diagnostic systems, OH, USA) and had a within-herd seroprevalence <5% (IDEXX Paratuberculosis Ab Test; IDEXX Laboratories Inc, Westbrook, ME, USA). A virulent cattle type MAP strain isolated from a clinical case (Cow69), with an identical IS900 – RFLP profile as the reference strain K10 (data not shown), which is the recommended strain type to use in experimental infections [13], was grown in supplemented 7H9 broth and used as inoculum. A high dose (HD) and a low dose (LD) inoculum were prepared (5×10^9^ and 5×10^7^ CFU, respectively), with both given on 2 consecutive days, corresponding to 5 times the recommended standard bovine challenge dose [13] and 10 times the lowest confirmed and consistent infectious dose for young calves [14]. In a first experiment, further referred to as the longitudinal study, serum samples were collected monthly and analysed from 7 animals experimentally infected with MAP at 2 weeks of age (5 HD and 2 LD) and from 6 non-infected age-matched controls for 17 months (approximately 500 days). In a second experiment, further referred to as the cross-sectional study, 35 calves were infected with MAP at 4 age ages (2 weeks and 3, 6 and 9 months) with 5 calves in each age group receiving a HD and 5 receiving a LD. Calves receiving the LD infection at 9 months after infection were not included in the analyses because the effect of this infection was expected to be low at 3 months post inoculation. Sixteen calves of the same age, housed under the same conditions, served as non-infected controls. Blood samples were drawn from the jugular vein when all cattle were exactly 1 year old. For logistical reasons, both experiments were conducted in two replicates, with equal representation of the different treatment groups in either run, with one run starting in February/March and the other in June/July. Samples were transported on ice, serum was harvested within 2 hours after sample collection and aliquots were frozen at −80°C. Animal care protocol M09083 covers the experimental infection of dairy calves with *Mycobacterium avium* subsp. *paratuberculosis* for the purpose of discovering biomarkers of infection by metabolomic profiling and was approved by the Health Sciences Animal Care Committee of the University of Calgary. Euthanasia was performed by injection of Euthanyl-Forte (pentobarbital 540 mg/mL) intravenously into the jugular vein.

### Tests to confirm exposure and infection with MAP

Sera were collected monthly from all calves and tested for antibodies using an ELISA (Pourquier ELISA; Institut Pourquier, Montpellier, France). Fecal samples were collected every month for MAP culture using para-JEM, TREK Diagnostic Systems (Cleveland OH, USA) and the presence of MAP was confirmed with IS900 PCR. Intestinal tissues were investigated for the presence of MAP by culture and IS900 PCR confirmation and lesions characteristic of MAP infection were determined on the basis of gross and histological pathology, as described [12].

Heparinized whole-blood samples were collected monthly by the Vacutainer system and transported to the laboratory in a thermos box (without cooling). At arrival, 2 to 4 h after sampling, 1.5-ml cultures were stimulated in 24-well culture plates (Greiner Bio-One Inc, Monroe, NC) with previously added Johnin (CFIA), PPDavium, and PBS (50 µl) and positive control stimulations with Pokeweed mitogen (Sigma-Aldrich, Oakville, ON, Canada). All Purified Protein Derivative (PPD) preparations and pokeweed mitogen (PWM) were added to a final concentration of 10 µg/ml. Cultures were incubated for 18 hours at 37°C and 5% CO_2_ in air. After incubation, plates were centrifuged and approximately 0.8 ml of supernatant was collected and frozen at −20°C until analysis. The IFN-γ contents of supernatants were measured in duplicate with a Bovigam ELISA kit (Prionics, La Vista, Nebraska, USA), in accordance with the manufacturer's instructions.

### Metabolite sample preparation

Serum samples were thawed in 3 batches and filtered twice using 3 kDa NanoSep microcentrifuge filters (which had been pre-washed) to reduce contamination. Filtrate was transferred to prewashed microfuge tubes; final sample volume ranged from 100-400 µL. Samples were brought to 650 mL by addition of D20, 130 µL of phosphate buffer containing dimethyl-silapentane-sulfonate (DSS, final concentration 0.5 mM), and 10 µL of 1 M sodium azide to inhibit bacterial growth. Final sample pH was adjusted to 7±0.05 by addition of HCl or NaOH.

### Spectrum acquisition

One-dimensional version of Nuclear Overhauser Effect spectroscopy (NOESY) spectra were acquired using an automated NMR Case sample changer on a 600-MHz Bruker Ultrashield spectrometer (Bruker Biospin Ltd., Milton, ON, Canada). The 1D NOESY pulse sequence had a water signal presaturation during relaxation delay of 1 second and mixing time of 100 milliseconds. Initial samples for each batch were shimmed to ensure half-height line width of approximately 0.8 Hz for the DSS peak at 0.0 ppm. Shims for each sample were then refined using a 1D gradient shim adjustment before spectrum acquisition. Spectra were acquired with 1024 scans, then zero-filled to 64 k points, followed with Fourier transformation. Additional 2-dimensional NMR experiments were performed for the purpose of confirming chemical shift assignments, including total correlation spectroscopy (2D ^1^H-^13^C TOCSY) and heteronuclear single quantum coherence spectroscopy (2D ^1^H-^13^C HSQC), using standard Bruker pulse programs. Original spectra are available for validation (upon request).

### Sample fitting

Processed spectra were imported into Chenomx NMR Suite software version 7.1 (Chenomx Inc., Edmonton, Canada) for quantification. Preprocessing in Chenomx Processor module took the form of Fourier transformation of FID signal with exponential multiplication by 0.2 Hz line broadening factor, water region deletion, and baseline correction using a manually calibrated spline mode to remove distortions of amplitude greater than background noise. Representative examples of spectra of samples from non-infected and infected animals are given in Figure S1. Metabolites were assigned based on comparison of both ^1^H and ^13^C chemical shifts and spin-spin coupling constants with those of model compounds in Human Metabolite Database [15] and Chenomx NMR Suite 7.1 software. Metabolites were quantified using the targeted profiling approach as implemented in the Chenomx software [16]. In total, 53 compounds were quantified in each spectra when signal to noise ratio was sufficient. These compounds were 2-aminobutyrate, 3-hydroxybutyrate, acetate, acetone, alanine, allantoin, arginine, asparagine, aspartate, betaine, carnitine, choline, citrate, citrulline, creatine, creatine phosphate, creatinine, dimethyl-sulfone, dimethylamine, ethanol, formate, glucose, glutamate, glutamine, glycerol, glycine, hippurate, histidine, isobutyrate, isoleucine, isopropanol, lactate, leucine, lysine, mannose, methanol, methionine, N,N-dimethylglycine, ornithine, phenylalanine, proline, propionate, pyruvate, serine, succinate, taurine, threonine, trimethylamine N-oxide, tryptophan, tyrosine, urea, valine, myo-inositol. Across all samples, profiled compounds comprised approximately 95% of total spectral area. All spectra were randomly ordered (within acquisition batches) for fitting in a Chenomx Profiler. Within each, compounds were profiled in order of decreasing concentration, refined from an average concentration/translation set derived from previous studies in mice. Each compound concentration was then normalized by dividing the measured concentration into the total concentration of all metabolites in that sample, excluding glucose and lactate, to adjust for dilution effects from sample preparation. Normalized metabolite concentrations were then mean centered and univariate scaled. Unknown metabolites were not tracked during data analyses.

### Statistics

To identify metabolites changing significantly with age within control and infected groups of cattle and between these two groups in the longitudinal study, the SAM method was used [17]. This is a well-established statistical method for identification of differentially expressed genes in microarray data analysis. In that regard, it was designed to address the false discovery rate (FDR) when running multiple tests on high-dimensional microarray data and is compatible with time-course data. The SAM assigns a significance score to each variable, based on its change relative to the standard deviation of repeated measurements. For a variable with scores greater than an adjustable threshold, its relative difference is compared to the distribution estimated by random permutations of class labels. For each threshold, a certain proportion of the variables in the permutation set will be significant by chance. The proportion is used to calculate the FDR.

Factors associated with the binary status (infected and non-infected) and confirmed by IFN-γ release assay were identified using multivariate regression analysis. Principal Component Analysis (PCA) was used to detect intrinsic clusters and outliers within the data set, followed by Orthogonal Projection to Latent Structures Discriminant Analysis (OPLS DA), and the bi-directional (X and Y) predictive OPLS-DA (O2-PLS) analyses using the SIMCA-P software suite (Umetrics, Malmö, Sweden). The OPLS-DA is an extension of PLS-DA, featuring an integrated Orthogonal Signal Correction (OSC) filter to remove variability not relevant to class separation [18]. The OPLS-DA yields a loading value for each metabolite (X-variable) and a score value for each sample, respectively representing a pattern of change correlated with a supervisory Y-variable (infection status or time after infection) and indicating the degree to which the pattern was present in each sample.

The quality of the models was evaluated with R^2^ and Q^2^, indicating the total variation explained in the data and the cross-validated predicted variation, respectively, using a default 7-fold cross-validation approach (which was also interpreted as the overall predictive ability of the metabolite profiling).

Score scatter plots, loading plots, and coefficient plots were generated in SIMCA-P. Also the ANalysis Of VAriance testing of Cross-Validated predictive residuals (CV-ANOVA) for assessing reliability of O-PLS models and the Receiver Operator Curves (ROC) were done using SIMCA-P.

Boxplot representation (*Stata Statistical Software: Release 12*. College Station, TX, USA: StataCorp LP) was used to visualize variation in levels of integrated compounds in control and infected samples.

## Results

The cellular immune response measured by the interferon-gamma release assay was used as a positive control for exposure. All infected calves had a strong reaction in the interferon-gamma release assay as soon as 3 months after infection and persisting for the duration of the trial. None of the control calves produced a positive result in this assay.

Two of the calves infected at 2 weeks of age with the high dose were continuously shedding MAP, with weight loss (likely related to the infection) near the end of the 17 month trial. These two cattle were euthanized at 472 and 485 days, due to animal welfare concerns. Consequently, the last monthly samples for those animals were missing from the longitudinal study analysis. Wherease these calves represent an unexpectedly high rate of fast progression to clinical disease, none of the other infected cattle demonstrated clinical symptoms indicative of JD.

Other indicators of infection identified in cattle that were part of the longitudinal experiment included fecal shedding (6/7), antibody positive on ELISA (5/7), gross pathology lesions (6/7) and MAP positive tissues (4/7). Cattle in the cross-sectional experiment had the following profiles: fecal shedding (24/35), antibody ELISA (15/35), gross pathology lesions (21/35), and MAP-positive tissues (17/35). Shedding was either intermittent, continuous or sporadic. Calves could not be categorized based on shedding pattern so that there were enough calves per category to perform a meaningful metabolomic analysis.

### Longitudinal study

To identify the earliest time after infection single metabolites or metabolite profiles can reliably predict MAP infection, longitudinal testing of infected and age-matched control cattle was performed. ^1^H-nuclear magnetic resonance (NMR) spectroscopy of serum was used to yield metabolite identification as well as quantitation. In this study, 7 infected calves at 2 weeks of age were compared to 6 controls for the duration of the 17-month infection trial. Monthly serum samples were analysed by ^1^H-nuclear magnetic resonance (NMR) spectroscopy. Separation of samples obtained from the 2 experiments was apparent by both PCA and OPLS analysis and was attributed to differences (Fig. 2).

The influence of developmental and diet changes during the 17 months of the life of the calves was assessed by performing SAM, on separate groups and between groups. The following metabolites increased significantly over time in both the infected and the non-infected groups: 2-Aminobutyrate, Alanine, Citrate, Hippurate, Dimethyl sulfone, Glutamine, Histidine, Isoleucine, Leucine, Lysine, Methionine, Ornithine, Pyruvate, Tyrosine, Valine. These metabolites represented the influence of developmental and diet changes during the 17 months of the life of the calves. Allantoin, Creatine, Isobutyrate, Tryptophan only increased significantly over time in the infected group, whereas Acetone, Isopropanol, Glucose, and Myo-Inositol only decreased significantly over time in the infected group (data not shown).

From the time-course analysis differences between groups, glucose was significantly higher for the duration of the experiment in the infected group, whereas Acetate, Dimethyl Sulfone, 3-Hydroxybutyrate and Methanol were lower in the infected than in the non-infected group.

To determine when the metabolite profiles started to differentiate between infected and non-infected cattle, samples were analysed by OPLS-DA for every single time point. Whereas R^2^ (>0.6) and Q^2^ (>0.4) values were obtained for respectively 7 and 5 individual time points, the CV-ANOVA p-value was generally high, with only the exception of p = 0.053 for 8.5 months post inoculation, corresponding with a R^2^ and Q^2^ of 0.682 and 0.445 (1 predictive component), suggesting overfitting of the models. This was supported by the fact that for almost half of the time points, no model could be generated.

**Figure 1:**
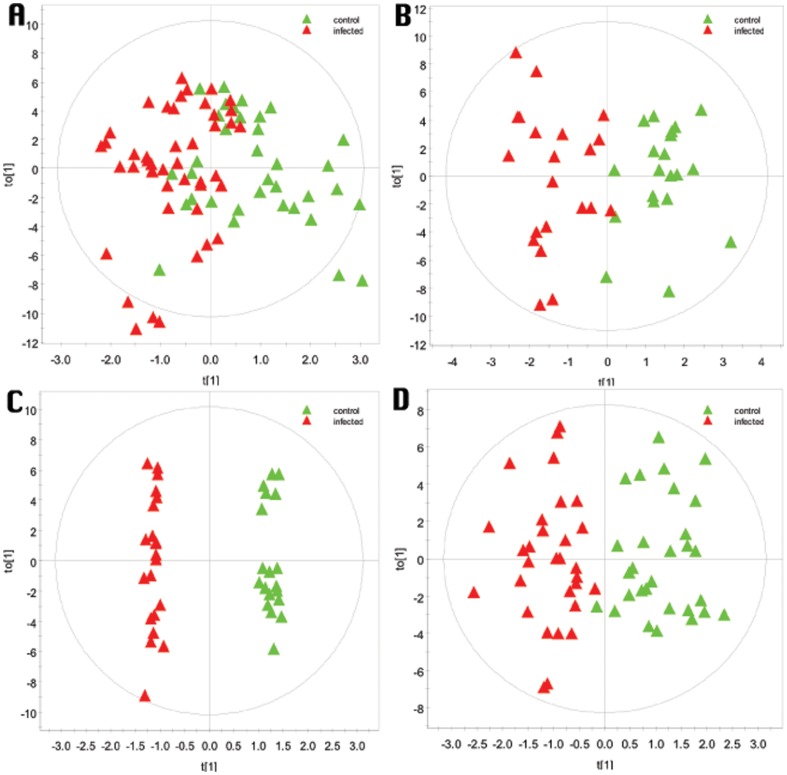
O-PLS-DA scores plots for different intervals after MAP infection for monthly serum samples of animals infected at 2 weeks of age compared to non-infected age-matched controls for a period of 17 months. A) <200 days after infection (R^2^Y  = 0.45, Q^2^Y  = 0.07; CV-ANOVA p = 0.50), B) 200–300 days after infection (R^2^Y  = 0.77, Q^2^Y  = 0.23; CV-ANOVA p = 0.15), C) 300–400 days after infection (R^2^Y  = 0.99, Q^2^Y  = 0.61; CV-ANOVA p = 0.09), and D) 400–500 days after infection (R^2^Y  = 0.78, Q^2^Y  = 0.37; CV-ANOVA p = 0.002).

Next, samples were divided in 4 age categories (<200 days, 200–300, 300–40,0 and 400–500 days of age), so that the categories contained respectively 6, 3, 3, and 4 samples, representing repeated measures over time for each animal. The corresponding OPLS-DA models are presented in Fig. 1. Separation between groups and clustering of groups along the X-axis was best achieved in the age category 300–400 days, based on examination of scores plots (Fig. 1) and corresponding R^2^(Y) values. The predictive capacity of the models as given by the Q2 value was also greatest in this time frame after infection. However, the CV-ANOVA only yielded a p-value of 0.09 for this age category, whereas the oldest age category that had the best validated model by CV-ANOVA (P = 0.012).

### Cross-sectional study

**Figure 2:**
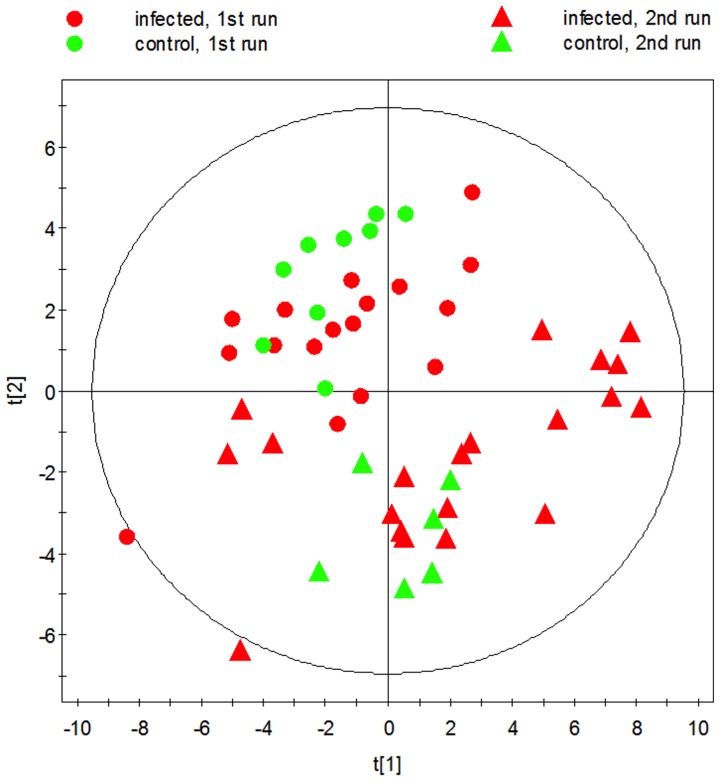
2D unsupervised PCA scores plot of infected and non-infected cattle at 12 months old. The clustering pattern between animals in first and second run can be seen.

The second study included sera from 35 infected calves (1-year old) with 16 age-matched control sera from non-infected calves. The infected group included calves at 12, 9 and 6 months after infection which had received either the HD or LD inoculum and calves only 3 months after infection with HD. All cattle were 12 months old serum collection (thereby avoiding confounding metabolic changes due to natural aging and developmental changes). Unsupervised principal component analysis (PCA) did not yield distinct separation between infected and non-infected cattle, but there was a separation between samples from the first and second runs (Fig. 2).

**Figure 3:**
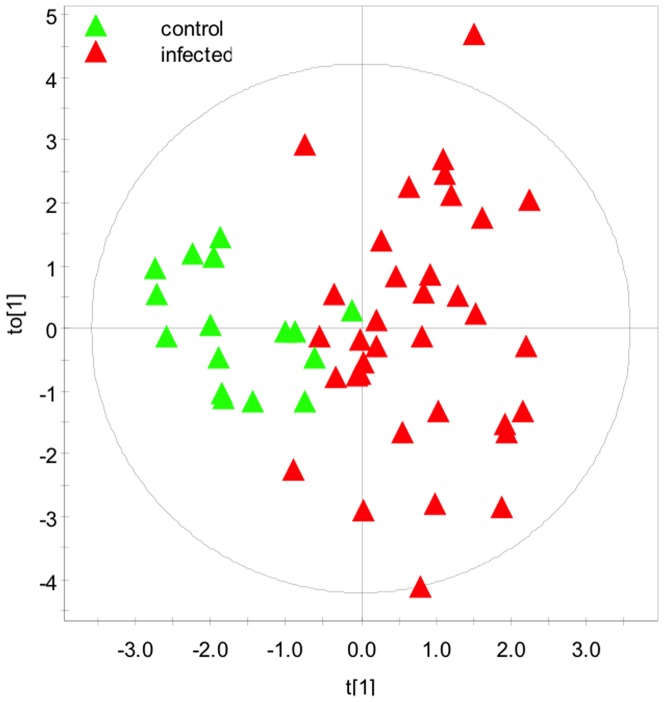
2D supervised OPLS-DA scores plot demonstrating the clustering pattern obtained for animals with known discrete infection status in dairy calves exactly 1-year-old dairy calves (R^2^Y  = 0.65, Q^2^Y  = 0.52). The metabolites were trimmed to the 16 most discriminating ones based on an analysis including all 53 metabolites.

When all 53 metabolites were included, an OPLS-DA model was created with R^2^(Y) of 0.757 and Q^2^ of 0.439 (1 predictive and 2 orthogonal components) and a CV-ANOVA p-value of 0.00018. From this analysis, metabolites were trimmed to those with most significant regression coefficients (n = 16) to create an improved OPLS-DA model. The scores plot from this analysis is shown (Fig. 3), revealing a statistical separation along the primary axis between control and MAP-infected cattle. This OPLS-DA yielded a R^2^(Y) of 0.645 and Q2 of 0.519 (1 predictive and 1 orthogonal components) and a CV-ANOVA p-value of 0.00000062.

**Figure 4:**
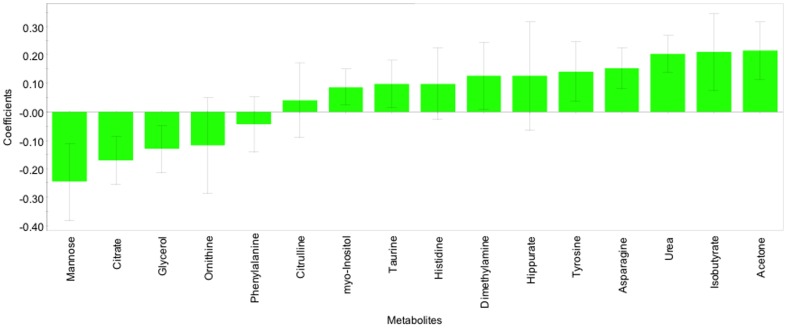
OPLS-DA coefficient plot of the 16 most discriminating metabolites; bars with negative values indicating metabolites that are significantly lower in MAP-infected than in non-infected and bars with positive value indicating metabolites which are significantly higher in MAP-infected than in non-infected animals exactly 1 year of age (R^2^Y  = 0.65, Q^2^Y  = 0.52). Only metabolites with a confidence interval that did not cross the zero line had significant changes (p<0.05).

The corresponding regression coefficients for the included metabolites, ordered according to their variable importance in the OPLS-DA model, are shown (Fig. 4). Serum mannose, citrate and glycerol concentrations were significantly lower in infected cattle, whereas acetone, isobutyrate, urea, asparagine, tyrosine, dimethylamine, myo-inositol were higher.

**Figure 5:**
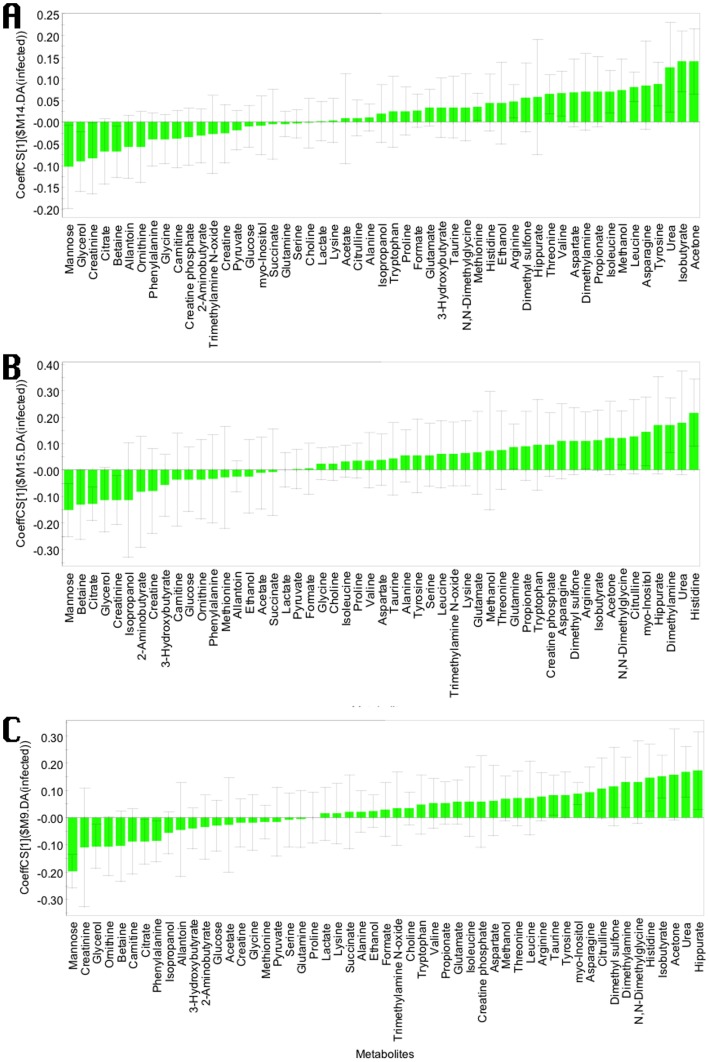
OPLS-DA coefficient plot of all metabolites; bars with negative values indicating metabolites that are significantly lower in A) non-infected versus high dose MAP-infected (R^2^Y  = 0.72, Q^2^Y  = 0.42), B) in non-infected versus low dose infected animal (R^2^Y  = 0.86, Q^2^Y  = 0.33), and C) non-infected against all MAP-infected animals (R^2^Y  = 0.76, Q^2^Y  = 0.44), and bars with positive value indicating metabolites which are significantly higher in those same groups of infected animals exactly 1 year of age. Only metabolites with a confidence interval that did not cross the zero line had significant changes (p<0.05). The negative coefficients indicate metabolites that are significantly lower in negative versus MAP-infected animals and vice versa.

Coefficient plots were also built for the OPLS-DA models generated to discriminate HD and control cattle or LD and control cattle (Fig. 5). More metabolites were increased than decreased in infected cattle. Mostly, the same metabolites were identified which contributed the most to these models compared to the model in which all infected cattle were grouped and contrasted against those that were non-infected. Most notably, mannose, creatinine, citrate and glycerol were decreased in the infected cattle in all models. Hippurate, histidine, dimethylamine, tyrosine, asparagine, urea, isobutyrate and acetone were increased in all models. Therefore, infected cattle behaved uniformly, regardless of inoculation dose (the model was not dominated by the HD infection group).

**Figure 6:**
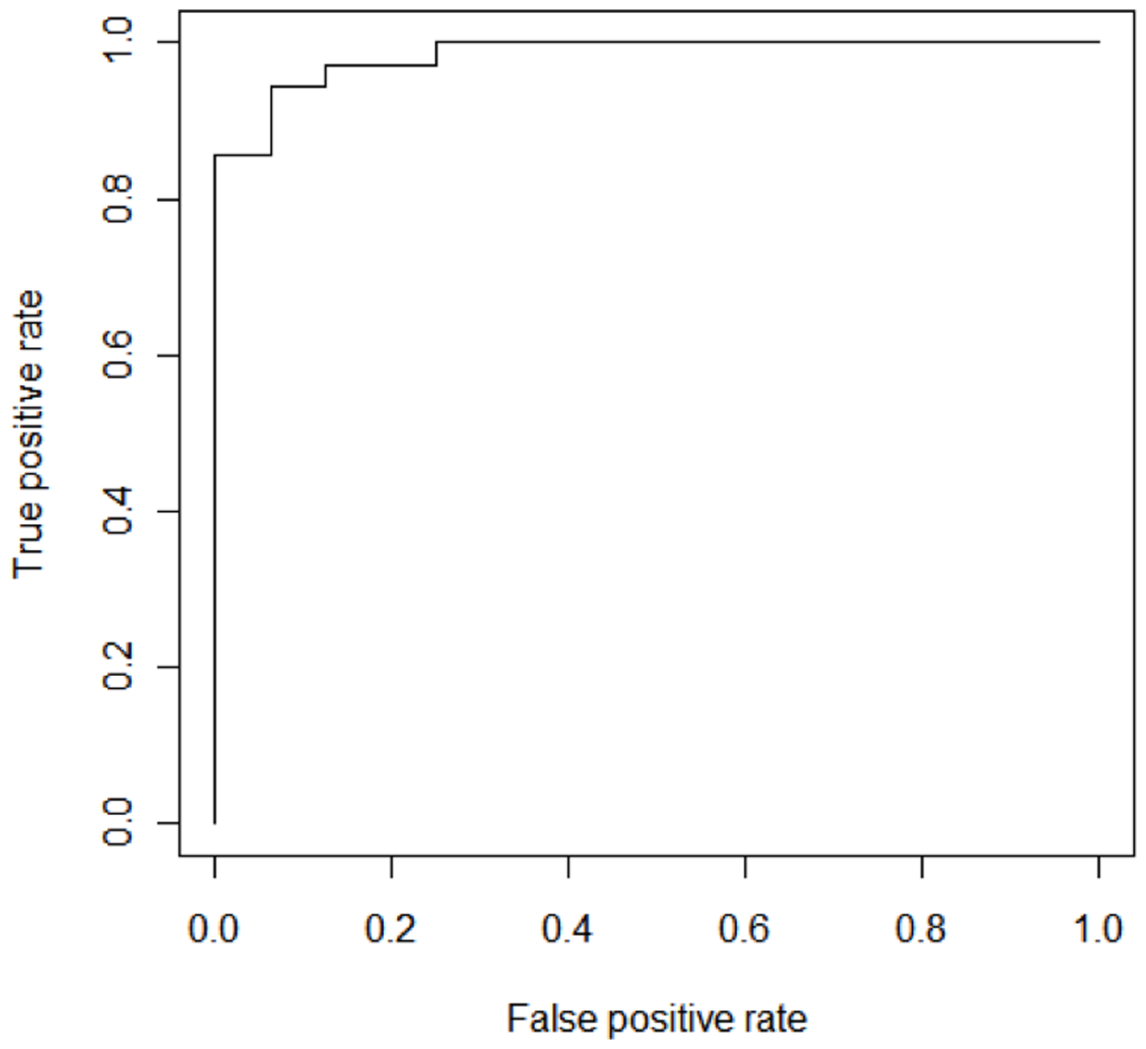
ROC curve for the trimmed O-PLS DA model including the 16 most significant metabolites. The AUC was 0.984.

Receiver operating curves were calculated for the OPLS-DA model, including all 53 metabolites and the model with the metabolites trimmed to 16 metabolites; the AUC were 1.0 and 0.984, respectively (Fig. 6).

**Figure 7:**
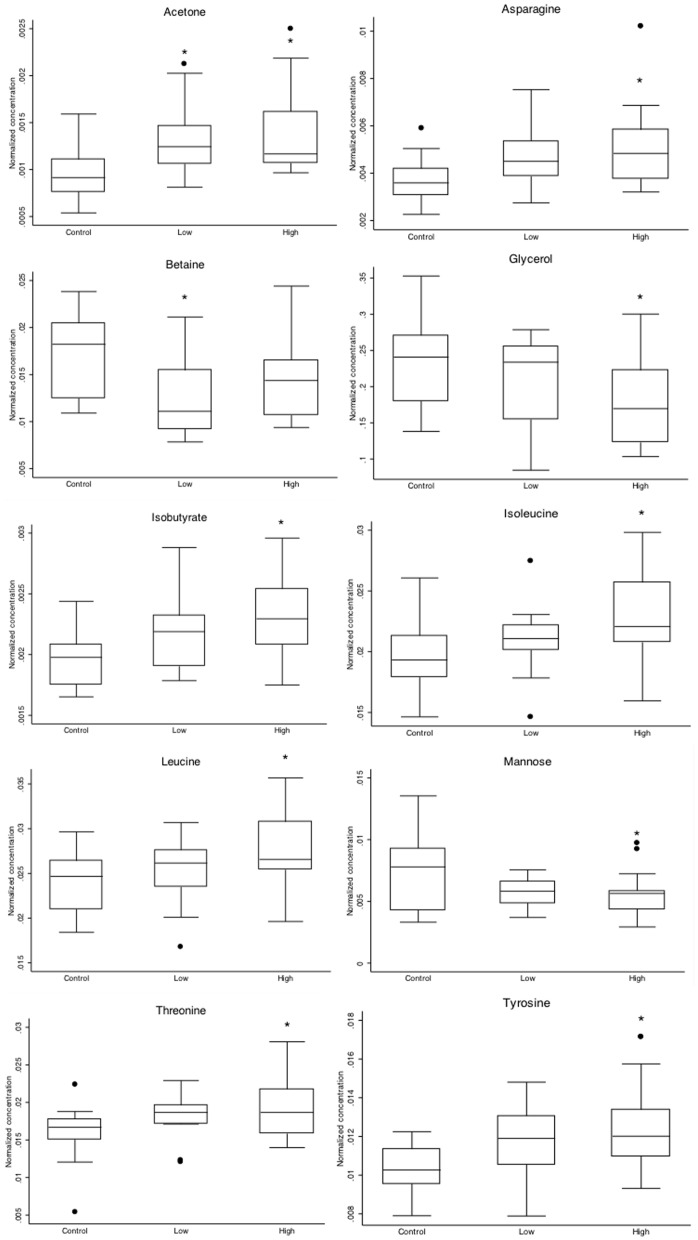
Boxplots of selected metabolites (acetone, asparagine, aspartate, betaine, glycerol, isobutyrate, isoleucine, leucine, mannose, threonine, tyrosine) for MAP-infected animals (n = 35) and non-infected age matched controls (n = 16). Significant differences with the control group by posthoc testing (Scheffe) after ANOVA are indicated by * (P<0.05).

Most of the metabolites incorporated in the final OPLS-DA model between all infected and non-infected cattle (Fig. 3 and 4) matched the set identified by the Student's *t*-test, without correcting for multiple testing between infected and non-infected cattle and also in one-way ANOVA between the non-infected animals and the dose groups. Boxplots of those metabolites are shown (Fig. 7). Betaine was missing from the metabolites which were lower in infected cattle and the amino acids leucine, isoleucine, threonine that significantly discriminated HD from control (Fig. 5 and 7) were also not represented in the final model as metabolites which were higher in infected cattle. Interestingly citrate, urea, dimethylamine and myo-inositol were part of the OPLS-DA model, whereas individually they did not significantly differentiate between groups.

Neither an OPLS (Y: the continuous variable time after infection being 12, 9, 6 or 3 months post infection), nor an O2-PLS analysis (Y1-time post infection; Y2: infection status) could be constructed which detected a significant influence of time after infection.

## Discussion

In the present study, NMR-based metabolomic analysis technology was used, apparently for the first time, to identify characteristics of the metabolite profiles of calves infected with MAP and to discover biomarkers to discriminate MAP-infected from non-infected controls. Despite the well known poor sensitivity of diagnostic methods, including the culture of tissues generally considered the gold standard, to detect MAP infection, especially in the early stages of infection, the combination of methods used in this study provided evidence of exposure and infection in the majority of the challenged cattle by positive interferon-gamma release assay, fecal shedding, antibody ELISA, Map culture of intestinal tissues, gross and histological pathology lesion scoring. The results of the longitudinal follow-up of the calves by these tests were previously reported [12], [19]–[21].

When samples from *Mycobacterium tuberculosis*-infected rats were analyzed with NMR [22], several tissue and serum metabolites related to membrane phospholipids, glycolysis, AA and nucleotide metabolism and antioxidative stress were altered. Similarly, analysis of serum from patients with leparomatous leprosy also resulted in metabolomics biomarkers such as certain polyunsaturated fatty acids and phospholipids, suggested to be useful for diagnostic purposes and assessment of disease progression [23].

This was apparently the first study to attempt identification of biomarkers of paratuberculosis in cattle experimentally infected with two doses and at several ages. Previously, studies have performed longitudinal metabolomics analysis on chronic infections, e.g. chronic wasting disease in elk [24] and *Schistosoma mansoni* infections in mice [25].

Good predictive models were created for experimentally infected calves at 1 year of age when fecal shedding was sporadic or absent and the presence of MAP-specific antibodies was infrequent. Therefore, signals were present which could be targeted to detect MAP infection before the cattle shed the organism or generated antibodies. Previously, cell-mediated immune responses [26] were identified early after infection and regarded as the only currently available test useful to detect infection in young stock [27]. Perhaps the metabolite profiles are the result of early immune responses [28], [29] and chronic inflammation [30]. Notwithstanding, it is also possible that physiological and pathological changes in the intestines soon after infection, changes in general metabolism, nutrient uptake and energy balance could have affected metabolite profiles.

Most surprisingly, metabolomic analysis of the 1-year-old animals revealed shifts in the concentration of several metabolites that were consistent over cattle infected for varying lengths and doses (which differed by 2 logs). Although the same metabolites discriminated LD and HD infected cattle from negative controls; therefore, dose did not affect metabolomic changes, it is noteworthy that immune-related outcomes are typically influenced by dose in parasitic [31], viral [32] and bacterial [33] infections. Moreover, whereas all treatment groups of this trial were purposely allocated into 2 independent runs, differentiation of infected and non-infected animals was still possible despite added seasonal variation to the metabolite profiles. This supported the idea that metabolomics analysis captures the general trends of a response and is less sensitive to variations. These findings are promising for application of metabolomics profiling to detect early infection under field conditions where the infection dose or time of infection is unknown and variable. The inclusion of animals at variable times after infection in the same analysis corresponded better with natural infection than the uniformity obtained by animals infected at exactly the same age. Therefore, this approach has the potential to identify differences in metabolite profile that are more likely to be sustained under field testing conditions.

Several metabolites (isobutyrate, acetone, and myo-inositol) helped to differentiate between infected and non-infected cattle in both studies. Although the direction of change of some of these metabolites (acetone, myo-inositol) was opposite in the two experiments, we inferred that increases and decreases in specific metabolites were indications of disturbances of the homeostatic concentrations of these metabolites as a result of the infection. In that regard, compensatory mechanisms can cause metabolites to oscillate around equilibrium concentrations.

Although biomarker models are not intended to explain the biology of the infection, some interesting associations between the identified metabolites and physiological changes likely occurring during MAP infection were noteworthy. For example, changes in concentrations of specific metabolites concentrations after MAP infection, such as acetone, citrate, glycerol and iso-butyrate, may have reflected altered energy metabolism in the inflamed gut. Conversely, significant changes in amino acid (tyrosine, threonine, isoleucine, leucine, asparagine) concentrations were regarded as important changes in protein turnover or deficiencies in infected cattle.

There were several commonalities in metabolite profiles between MAP infection and other chronic diseases, such as IBD [34], [35], various cancers [36] and diabetes [37]. Although the same metabolites were affected, the direction of the change often differed. As discussed earlier, common imbalances in key metabolic pathways were identified, but depending on the chronicity and time of sampling, these imbalances might be contradictory. Although in this study metabolite profiles were investigated long before clinical symptoms occurred, our findings matched further progressed stages of infection in which the body has compensated for deficiencies or has exhausted stores.

There was evidence of increased demand for overutilization of amino acids, likely associated with reduced absorptive capacity of the inflamed gut, a hallmark of JD [38], [39]. This was comparable with elevation of several amino acids (tyrosine, tryptophan, threonine, and isoleucine), which accompanied muscle wasting in human cancer patients [36].

Acetone, a secondary energy source in the absence of glucose, was the single most discriminatory metabolite in MAP-infected cattle. To spare glucose by providing a substitute energy source, glucagon stimulates degradation of fatty acids and conversion of surplus acetyl CoA to ketone bodies (including acetone). It is only when glucose sources are severely restricted that excess ketone bodies are produced. The higher level of acetone in MAP-infected cattle was consistent with an energy deficit and a higher mobilization and degradation of fat stores. Metabolomic profiling of the serum DSS-induced Ulcerative Colitis (UC) demonstrated increases in ketone bodies, such as acetoacetate, acetone and 3-hydroxybutyrate and decreased glucose concentrations, reflecting the high demand of the body for energy [34]. Ketone body concentrations were also markedly increased in septic rats, especially in non-survivors [40] and in mice intraperitoneally infected with *Staphylococcus aureus* [9]. Their increase might be related to enhanced fatty acid oxidation. As the major source of energy, fatty acid oxidation was significantly enhanced to meet the energy requirement [41]; therefore ketone bodies accumulated.

It is noteworthy that the incidence of negative energy balance resulting in ketosis is high in the first months of lactation in dairy cows [42]. This frequent occurrence will undoubtedly jeopardize any possible detection of ketone bodies as an indication of MAP infection in lactating animals. Regardless, detection of acetone levels in the dry period could circumvent this problem. Previously, there was no apparent correlation between shedding of MAP and subclinical or clinical ketosis as measured by beta-hydroxybutyrate concentrations [43].

Iso-butyrate and the branched chain amino acids (BCAA) leucine and isoleucine concentrations were increased, whereas citrate concentrations were decreased in MAP-infected calves. This was in agreement with some of the most important metabolite changes in rats in the fasted state after being fed a high protein diet before they were fed a high fat/high sucrose diet [44]. Both the LD and HD infected groups had a significantly lower body weight than the control group (data not shown), suggesting that energy intake and consumption were affected by the intestinal inflammation [12]. The main contrasting finding was that mannose belonged to the metabolites increased in this study, whereas it was decreased in MAP-infected calves. This discrepancy might be signifying the difference between energy and metabolomic state versus the effects of MAP-infection and inflammation. Mannose binding protein (MBL) is upregulated after infection with *M. tuberculosis* [45] and during other acute phase responses [46]. A dramatic increase in MBL might explain the decline in free mannose in the blood. Concentrations of BCAA and tyrosine were also increased in obese subjects compared to lean controls [47].

Altered concentations of amino acid in the cross-sectional study were consistent with JD as a protein-losing enteropathy [39], [48], [49]. It is noteworthy that JD is often compared with Inflammatory Bowel Disease (IBD) in humans, due to pathological similarities, because of a conspicuous overlap between genes predisposing for IBD and involved in host responses to mycobacteria [50] and because several reports of detection of MAP in samples obtained from IBD patients have lead to the conclusion that an association between MAP and IBD cannot be excluded [51]. In one study, Crohn's disease (CD) and Ulcerative Colitis (UC) had similar impacts on blood isoleucine concentrations as detected in this study [35], as was the case in a mouse model of UC [52], whereas in other studies it was slightly reduced [53] or unchanged [54]. Citrate concentration was decreased in these first two mentioned studies, analogous to this study. Interestingly, in serum and plasma of IBD patients, opposite to our MAP-infected calves, mannose and formate concentrations were significantly increased, whereas urea was decreased [35]. In that regard, the substantial increase in urea concentrations in MAP-infected calves was interpreted as increased muscle turnover, likely to compensate for reduced AA and energy intake.

## Conclusions

Metabolomic analysis yielded a clear separation between non-infected and MAP-infected groups, indicating a substantial imprint from the infection on the metabolism of calves during the early stage of the disease. In a longitudinal follow-up study, the strongest separation occurred around 12 months after infection, wheras a cross-sectional study demonstrated that at 3 months after inoculation the same characteristic profile was found as at 12 months after inoculation. Limited differences were identified between HD and LD, signifying that effects were both specific and dose independent. That cattle at various intervals post-infection had similar changes was strong support for diagnostic potential of the metabolite profiles. Altered metabolomic pathways included changes in AA metabolism, biosynthesis and degradation, ketone body synthesis and degradation, and TCA cycle activity.

Although biomarker discovery using experimental infections has the advantage of documenting when the biomarker detects the infection, validation of the use of metabolomics to detect infection under field conditions will need to be done to ultimately demonstrate its utility.

## Supporting Information

Figure S1 **Representative 1H NMR spectra of serum samples from non-infected (A) and MAP-infected cattle (B).**
(TIF)

Data S1 **Metabolite concentrations of all the samples included in the longitudinal and the cross-sectional studies.**
(XLSX)
