# Supplementary figures and images for: Metabolomic Profiling in Cattle Experimentally Infected with Mycobacterium avium subsp. paratuberculosis
Source: PLoS One. 2014 Nov 5;9(11):e111872. doi: 10.1371/journal.pone.0111872 (PMC4221196; doi:10.1371/journal.pone.0111872)

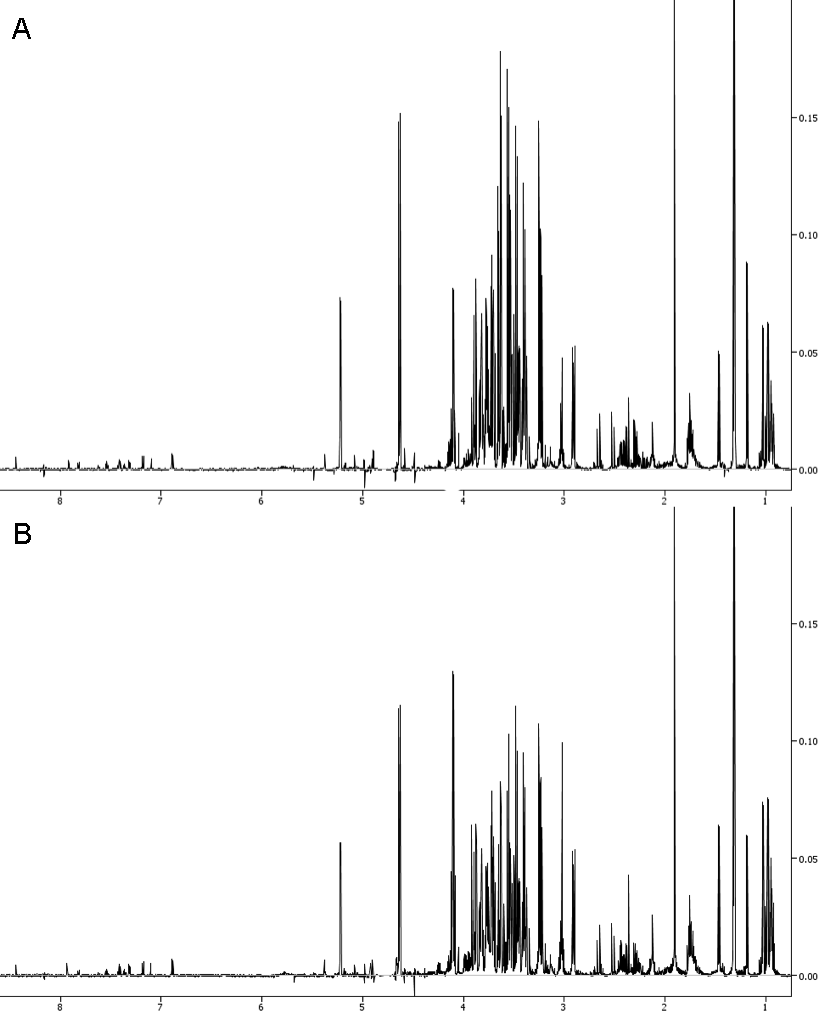

Supplement: Figure S1 — Representative 1H NMR spectra of serum samples from non-infected (A) and MAP-infected cattle (B). (TIF) [file pone.0111872.s001.tif]
